# Stories from a life studying circadian rhythms and sleep

**DOI:** 10.1093/sleepadvances/zpad040

**Published:** 2023-12-10

**Authors:** Charmane Eastman

**Affiliations:** Biological Rhythms Research Laboratory, Department of Psychiatry and Behavioral Sciences, Rush University Medical Center, Chicago, IL, USA

**Keywords:** shift work, jet lag, light history, melatonin, winter depression, spontaneous internal desynchronization, two-process model of sleep, night owl, NASA Space Shuttle, light treatment

## The Unlikely Path

In Brooklyn, in junior high, I was in a program that did grades 7, 8, and 9 in 2 years. In high school, I was sent to an after-school program for gifted underachievers. I chose to go to Albany State Teachers college (now called SUNY Albany), not because I wanted to be a teacher, but because I could go to any New York state college with free tuition from a Regents scholarship, and Albany was far enough away from my parents. They wanted me to live at home and go to college in Brooklyn. My major was math, and my minor was physics. I especially liked the physics classes, where I was the only girl. I got the best test scores of anybody in one math class, but the teacher gave me a B because I hardly ever came to class. It was at 9:00 am, way too early for me, an extreme night owl since childhood.

I took an introductory course in psychology and got hooked. I ended up taking as many courses in psychology as math. I was planning to change my major to psychology. When my father found out, he was not pleased. He (who was born in 1895) said that psychology is not a real science. And in any case, first you have to learn to cook and clean and be a good wife and mother.

After college, I moved to Boston and tried to get a job related to psychology. I interviewed for a lab tech job with Charles Gross at Harvard who studied vision in monkeys, but I didn’t get the job. I ended up working a boring job for a company that made interferometer spectrometers. A few months later, Charlie called me and offered me the job. The girl he had hired instead of me made a bad mistake and got fired. During surgery on a rhesus monkey to make a lesion in one part of the brain involved in vision, she handed them the pan of alcohol instead of the saline, so the lesion was much bigger than planned. I ended up loving the lab tech job, which included 3 hours per day of running monkeys in visual discrimination tests with a Wisconsin box. Nowadays, computers do it. The job with Dr Gross was the start of my 9 years as a lab tech at Harvard, MIT, and the University of California at Berkeley. I worked in four different labs with four different bosses and helped with experiments on rhesus monkeys, rats, cats, rabbits, and one piglet. I also vaguely remember hamsters and frogs. I learned a little about the visual and auditory brain systems. I learned to do histology on brain tissue, and especially liked making Golgi stains that showed neurons with their tree-like dendrites. But what I really learned the most was about how graduate students, postdocs, and their mentors interacted. The graduate students and postdocs would complain to me (in confidence) about their mentors and vice versa.

People started saying that I should go to graduate school. But I said, “Why? I like my job.” I was a super tech. I knew that I couldn’t be a graduate student because you have to talk in seminars and give talks. In all 4 years of college, I never raised my hand once to speak. Because it was a teachers college, there was a required course in which you had to give a speech on a topic of your choice. I put it off for a few years, but finally, because otherwise I wouldn’t graduate, I took the course. In order to give my speech, I had to go the local bar and have some vodka and coke before class.

After 8 years of being super tech, and a little behavior therapy and assertiveness training, I decided to apply to graduate school. In those days, you got printed brochures and application materials. I made three piles: not good enough for me, too good, and too prestigious for me (I only had a B average in college), and OK to apply. I got accepted by Wilse B. Webb at the University of Florida in Gainesville. He studied sleep and circadian rhythms. I had to go to Florida for my grandmother’s funeral, so I asked Webb if I could visit. Otherwise, I would have just showed up when the semester started. He showed me around and after a while he said that the only place that would be better for you would be with Alan Rechtschaffen at the University of Chicago. But I explained that I didn’t apply there, and it was past the deadline. (It was in my too good for me pile.) He said that I should just write a letter to Rechtschaffen, so I did. I got a long letter back from Al, describing all the different experiments in sleep that they were doing, and all the different animals they were studying, including humans.

## Alan Rechtschaffen, PhD, and His Sleep Lab

That visit with Webb led to my 9 years living at Al’s sleep lab, from 1974 to 1983, 6 years as a graduate student and 3 as a postdoc. I say “living” because that’s what was expected of us, to be working there day and night. Since the lab was in what used to be apartments with kitchens and bathrooms, it was actually possible to live there. I had my own setup with two little rooms, one with four cages, one for each rat, and one for the old EEG machines which ran 24 hours/day. I recorded temperature (from thermistors implanted over the brain), sleep and wake (with a computerized sleep scoring system that could tell REM from quiet sleep) and activity (from a tilt cage). A few years after being there, we got a word processor, so we didn’t have to literally cut with scissors and paste with scotch tape to work on publications. Al took all of us to the annual sleep meetings, to APSS, then called the Association for the Psychophysiological Study of Sleep. I think the first one I went to was in 1974 or 1975. There were no trainee days, no trainee grants, no special courses or events for trainees. But the meetings were very small, and we trainees found each other to hang out with. Al introduced me to Michel Jouvet and William Dement and maybe others. I was overwhelmed. If you’ve ever seen I Love Lucy episodes in which she meets famous movie stars and acts all starry-eyed and tongue-tied, then you get the picture. NIH grants were also different in those days. Each of us got to write a page with what we had done and what we proposed to do. Al only wrote one grant at a time, renewing it each time, and it sustained the lab for decades.

Al was my mentor, of course, but the biggest gift he gave me was the freedom to pursue my own interests. After our first publication together [1], everything else was about circadian rhythms. When I was still a relatively new graduate student, I gave him an abstract that I wrote for the annual Sleep meeting. When he returned it to me, the only changes he had made were to add a few commas, and to cross his name off. I was shocked. Why didn’t he want to be an author? Was it that bad? Was he mad at me? I finally gathered enough courage to ask him about it. He said that he really didn’t have enough to do with the work to claim authorship, but that he thought it was good—except of course for the commas. Whew! What a relief! And what fairness. What honesty. What integrity. Looking back on my CV, I can see several abstracts from his lab without his name on them.

This old-fashioned academic custom was not destined to last. Sometime later, Al called a meeting in the lab library. His demeanor was a little unusual, as he prepared to tell us something grave and important. He seemed apologetic, almost shy. Here was the story. In the last review of the renewal for his Research Career Development Award, he was told that he didn’t have enough publications, and that he should put his name on all the papers written by his graduate students. This is what all lab directors did, and so should he. Or something like that. I don’t remember exactly. But I do remember that without hesitation we all chimed in—Of course! It’s only right! We all wanted his name on our stuff anyway. We were proud to be associated with him. He seemed so sweet that day.

A few years after I was there, we got a computer. It had tall, large cabinets with reel-to-reel tapes on the top that jerked back and forth. There was a teletype with paper with holes on each side that rolled up with each line. There was no screen. I learned to program with BASIC and got addicted to the process. I was writing my dissertation “Circadian rhythms of temperature, waking, and activity in the rat: dissociations, desynchronizations, and disintegrations,” on what happened to rats after a 12 hours shift of the light/dark (LD) cycle, in different LD cycles, like 4 hours, 22 hours, and 28 hours days or constant light (LL). It was more fun to make models of what happened on the computer than to write. I didn’t know it then, but it turns out that over the course of my career I used many similar LD cycles on humans (9 hours advance shifts, 9 hours delay shifts, 12 hours shifts, 4 hours, 5 hours, 22 hours, and 26 hours days).

## Spontaneous Internal Desynchronization, the One Circadian Oscillator Model, and the Two-Process Model of Sleep

**Figure 1.**
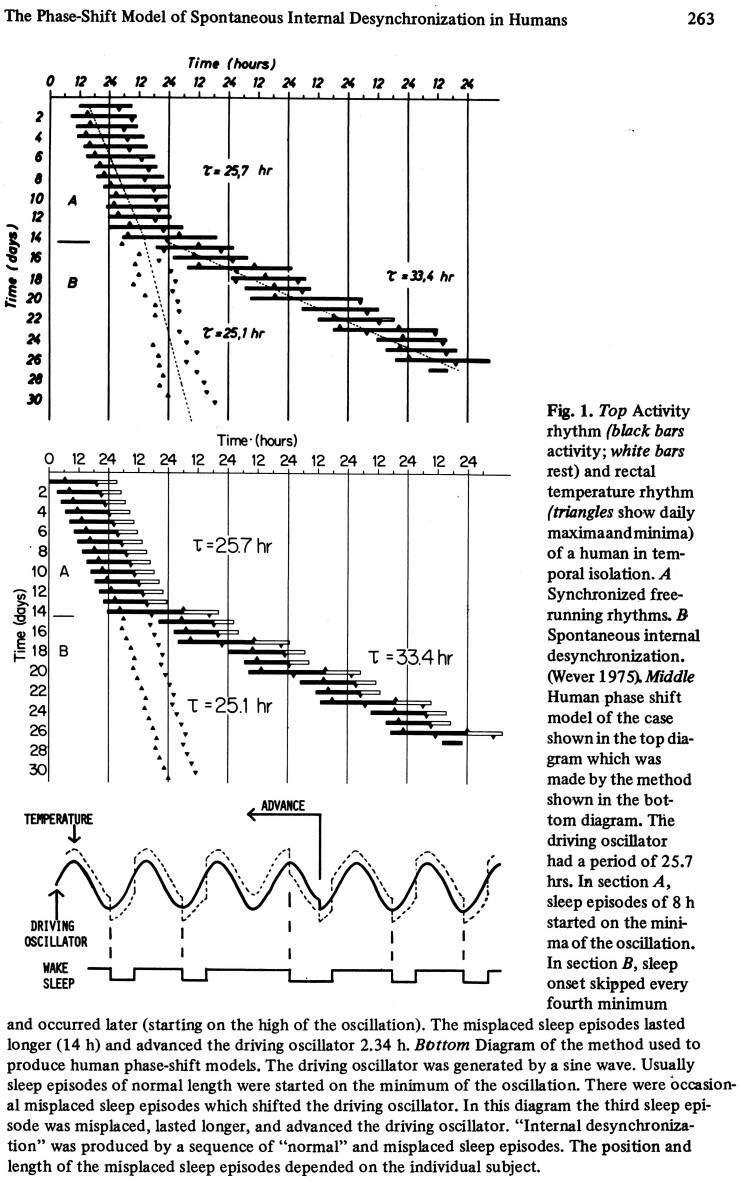
A page from the chapter by Eastman in the book edited by Aschoff et al. [2] showing an example of spontaneous internal desynchronization from a subject who lived in the underground bunker and the computer model, which mimics it using a single master oscillator.

In graduate school, I became fascinated by spontaneous internal desynchronization which happened to some subjects who were put into temporal isolation and lived for weeks in apartments that were in underground bunkers at the Max Planck Institute in Andechs, Germany. An example is shown in Figure 1 [2]. The top shows a typical case of spontaneous internal desynchronization of a subject from the bunker. In part A, the temperature and sleep–wake rhythms free ran synchronized together. Note that the body temperature minimum and maximums are represented by triangles. Later, I learned that these times were chosen by “the triangle lady” at the Institute by looking at the raw 24 hours rectal temperature recordings and drawing in triangles at those spots. In part B of Figure 1 top, the temperature rhythm continued to free run with a circadian period, but the sleep–wake rhythm ran with a period of 33.4 hours. Cases like this led Wever and Aschoff [3, 4] to postulate that human circadian rhythms are controlled by two oscillators, one for internal rhythms such as temperature, and one for the activity-rest (sleep–wake) rhythm. This theory was accepted, written up in text books, and endorsed by Kronauer and Czeisler [5].

Continuing to play on the computer instead of writing up my dissertation, I made models that mimicked several cases of spontaneous internal desynchronization like the one shown in Figure 1 middle. This showed that the pattern could be produced by a single master oscillator. I added this model to the end of my dissertation.

Alexander Borbely invited me to talk at a symposium at the Congress of the European Sleep Research Society to be held in Amsterdam in 1980. This was my first trip out of the US, and the first time I presented my one oscillator model of spontaneous internal desynchronization in humans [6]. I sent a letter to Aschoff, asking if I could visit, since I was going to be in Europe. I can’t believe that I did that. He had never heard of me or my advisor, Rechtschaffen, but was gracious enough to invite me anyway. I had brought lots of printouts of figures from my rat data and the human computer models. Aschoff and Wever and a few others gathered around my computer simulations of spontaneous internal desynchronization and talked to each other in German and mostly ignored me except for an occasional question. I didn’t understand what they were saying, but they seemed both annoyed and perplexed by me. I was dumbstruck by being in the presence of the famous Aschoff and Wever and could barely talk. Nevertheless, I got invited to give a talk at Aschoff’s Ringberg meeting which happened a couple of weeks later.

Alex Borbely and Serge Daan, among others, were at Aschoff’s famous Ringberg meeting. After my talk and Borbely’s, Serge was very excited and was drawing what would become the human two process model on a blackboard. The meeting led to many collaborations between Daan and Borbely and Beersma about the two-process model. In a book chapter [7] when discussing spontaneous internal desynchronization, they wrote “Eastman (21; and this volume) stated that the assumption of a second oscillator is not required to explain these observed patterns.” Ref 21 is my PhD dissertation. They wrote in the acknowledgments “The idea of our approach originated at the Ringberg Conference on Structure and Physiology of Vertebrate Circadian Systems in response to stimulating papers by Drs. Eastman, Borbely, and Zulley.”

After the Ringberg meeting, I decided to send my one oscillator model paper to the journal Science. It turned out to be the first and last time I submitted anything to Science. I sent a draft to Aschoff and asked if he would read it or give it to someone else to comment on. He gave it to Serge Daan. Serge sent me a long typed two-page letter that was full of stinging, insightful, and complicated criticisms of what was missing from my model. But he did say “As you will recall from the Ringberg meeting, I think your idea that human ‘internal desynchronization’ does not have to be based on two separate driving oscillators is quite interesting.” And he also said “The idea occurring to me now after reading your paper, is that we could instead make a joint manuscript for Science. We could then refer to two sources where a) the original idea is presented (your paper for the Ringberg symposium) and b) the implications of a series of model simulations . . . fully expounded . . . .” I wrote back that I was flattered and delighted at the invitation, but that I wanted to try to publish my Science paper first, and I hoped that we could still work on a joint manuscript with his refinements added later. I was thinking that if my name and his were on a paper, then people would think it was mostly his (the big shot famous person) and not mine (the little no one). Of course, my Science paper did not get accepted. In 1982, Serge sent a paper to Science with me and Beersma as authors. The title was “Single oscillator model of human sleep and wakefulness: Circadian gating of a renewal process.” It didn’t get accepted either. In a 2010 paper, the History of Chronobiological Concepts [8], Serge wrote about the “conundrum of internal desynchronization.” I think he meant that having an activity oscillator with very long or short periods, outside of the circadian range, was a problem for the two-process model of sleep. He wrote “The leading authorities in Andechs and in Harvard considered this proof of two separate pacemakers one controlling temperature, the other sleep and wakefulness … At the conference in Ringberg, Charmane Eastman pointed out the weakness of this argument……This led Borbely and Daan to develop the two-process model of sleep…..with a single circadian pacemaker that gates a homeostatically controlled sleep process.” P18.

The last paper I wrote about my model was a chapter in a 1984 book edited by Moore-Ede and Czeisler [9]. I clarified that the single oscillator may itself be composed of multiple oscillators such as seen in isolated mammalian organs and tissues. I also described how the phase relationship between the circadian oscillator and sleep differs depending on whether it is a free run or entrainment to a 24-hour day or to a 26 2/3-hour day. If you are a new researcher and are actually reading this, then remember if you have a good experiment or theory to publish try to get it in a journal first and not a book chapter.

## Rosalind Cartwright, PhD, and the Sleep Center

In 1983, Cartwright hired me as faculty in the Psychology Department at Rush University Medical Center in Chicago. She didn’t want me because I wasn’t a clinical psychologist, but she did want somebody who could write grants. Al Rechtschaffen encouraged her to hire me for that reason. I was called the laboratory coordinator, or lab director, of the Sleep Disorders Service and Research Center that she had established. She was also the Chairman of the Psychology Department which was a rare stand-alone department in a medical center. After she retired, and one new chairman who got fired, it was swallowed up into the Department of Psychiatry & Behavioral Sciences.

Cartwright was incredibly busy directing the sleep clinic, the psychology department, and her own grants on sleep and depression. She gave me every job she could possibly think of to give me to lessen her load. I was basically her assistant. It took me a long time to realize that I couldn’t possibly do everything she asked, and that I had to choose what was most important. I learned how to see patients in the sleep clinic. It was truly fascinating until I finally got tired of sleep apnea. She made me get an Illinois State Psychology License, and I had to study for many nights to take the exam. I passed easily and my hours in the sleep clinic made me eligible. It turns out I didn’t need that license for anything I ended up doing; it was just a waste of time.

My favorite part of being Ros’s assistant was helping her with her study of divorced people and sleep [10]. My job was to categorize them as major depression, or not depressed or other categories according to the Research Diagnostic Criteria (RDC), which were used until the DSM-III took over. I had learned about these during my postdoc supported by a training grant in the Psychiatry Department. After an hour of telling their stories and usually crying in her office, she sent the divorced people to me. I heard a lot more than I needed to categorize them, but they were all loosened up and I couldn’t stop them from talking. I learned that many people are depressed after divorce, even if they were the ones who wanted it. To many it was a complete surprise. I would go home to my boyfriend and ask: “How are we?” instead of “How are you?”

## The Biological Rhythms Research Lab—Beginnings

In 1984, I got an in-house grant to study circadian rhythms in humans. With this money ($14 252) I bought an Apple IIe computer and a portable Vitalog device to collect body temperature once a minute from a rectal probe. My first paper [11] describes the sleep and temperature rhythms of my first two subjects, me (called CIE in the paper) and MEJ. We followed a 26-hour schedule (bedtime and waketime 2 hours later each day) for several days. We used black vinyl to cover the bedroom windows, to make it very dark. The data from the Vitalog monitor were fed into the Apple computer. My temperature rhythm entrained to the 26-hour day, which did not surprise me as I am an extreme night owl. I would have been happier living on the space station in Star Trek Deep Space 9, which runs on a 26-hour day than in the 24-hour day currently produced by planet earth. While on the 26-hour day, I was actually very sleepy at bedtime and woke up alert just like a morning person, all as predicted by oscillator theory. Quite a change from not feeling at all sleepy at bedtime, taking a long time to fall asleep, and waking up sleepy and groggy. Actually, I would have been happy on a 25-hour day, because 26 was just at my limit of entrainment. Subject MEJ did not entrain to the 26-hour day and had a lot of trouble following the 26-hour sleep schedule after the first 3 days on that schedule. A later paper [12] included two more subjects along with CIE and MEJ. One of these entrained and one did not.

With this pilot data, I obtained an NIH New Investigator Award (R23 NS23421) with 3 years of funding from 1986 to 1989. The first year’s direct costs were a whopping $31 917! That grant turned into an R01 and went from year 4 to 13 and ended in 1999. My first paper supported by this R01 [13] again explored a 26-hour day. But this time, some subjects received bright light from light boxes for 2 hours in the evening before bed and stayed in dim light for 6 hours after waking, either by staying indoors or wearing very dark goggles if they had to go outside. Most of the subjects given evening light entrained to the 26-hour day, despite the presence of the conflicting 24-hour zeitgebers. This was the start of a series of studies to determine how circadian rhythms could be shifted in subjects living in the real world, which would lead to schedules to help night shift workers and for preventing jet lag.

Usually, studies on phase shifting and the limits of entrainment were done in temporal isolation units or in hospital suites where subjects lived for many days. Our studies were field studies because I didn’t have such a lab. This forced us to develop methods to keep track of subjects who were living at home. We had them call an answering machine before bed and after waking to confirm times on their sleep logs. We took the wrist bands off of some activity monitors and made them into necklaces to sit the sensor on the chest to get a better reading of light intensity at eye level. We learned the best ways to make bedrooms dark and found the thickest black plastic. We discovered black masking tape to cover holes. We even put air conditioners in the windows if subjects had to sleep during the day when it was hot. We acquired different types of light boxes, used as many as three big ones for a subject and lugged them to the subject’s homes. We bought various dark sunglasses and goggles for when subjects needed to avoid bright light. We developed mathematical demasking techniques, to reveal the endogenous circadian component of the temperature rhythm, which is otherwise masked by activity.

We studied 12-hour shifts of the sleep schedule with different patterns of bright light and with blocking out bright light with dark sunglasses to facilitate circadian rhythms phase shifts as in DN [14] and SDN [15]. We usually used three letters to designate each set of studies, and I am using them here for those from my lab who may be reading this. We compared the duration of bright light (3 or 6 hours) with 12-hour shifts in LDN [16], 9 hours advance, and 9 hours delay shifts with facilitating or conflicting bright light, ADN [17], 10 hours delay shifts with different intensities of light, low (<250 lux), medium (~1230 lux), and high (~5700 lux), BDS [18] and a pattern of intermittent bright light (40 min of bright light alternating with 20 min dim light) with 9 hours delay shifts of the sleep schedule, EDN [19]. All these studies and more were supported by my first R01.

## NASA Space Shuttles—The Light Stuff

One day in 1991, out of the blue, I got a call from Richard Jennings, MD, Deputy Chief, Medical Operations Branch of NASA’s Space Center in Houston. It took me a while to figure out what he was talking about. He was asking me if I would make schedules of light and sleep for the astronauts to follow before their space shuttle missions. These missions required the astronauts to follow sleep schedules radically different from their usual schedules, making them shift workers in space. I said—Isn’t Czeisler doing this? He said—Not anymore. I never found out why, but of course I said yes.

**Figure 2.**
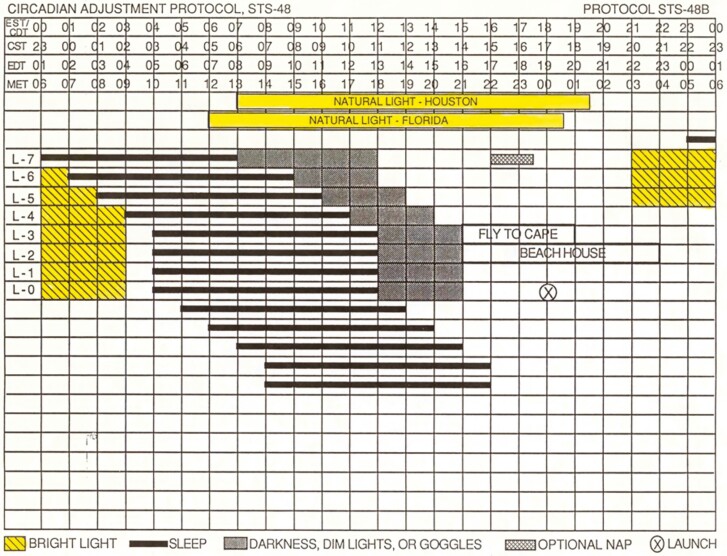
A schedule made by Eastman and Stewart to shift the circadian rhythms of one team of NASA astronauts before the launch of space shuttle STS-48. The top has four timelines showing the times in Houston (central time) and Cape Canaveral (eastern time) in standard and daylight-savings times. The fourth timeline is MET or Mission Elapsed Time which starts at the time of launch (00). The yellow rectangles under the timelines show the time of daylight for the two places. Black bars show the prescribed sleep times. Sleep was delayed by 1 hour each day for the first 4 days. Yellow shows when they should be exposed to bright light. The gray areas show when they should avoid bright light.

The schedule that Charles Czeisler, MD, PhD, and his coworkers used can be seen in figure 2 of their publication [20]. After sleeping at a normal time on the seventh day before the launch, there was a large abrupt delay shift of the sleep schedule. The astronauts had to go to bed about 9 or more hours later and sleep during the daytime. Bright light was applied around the time that they ordinarily slept, from around midnight to 7 or 8 in the morning. Lights (390 fluorescent lamps) were installed on the ceiling of the conference room in the astronaut’s crew quarters in Houston producing 10 000 lux. The last 2 days before the launch were spent in crew quarters at the Kennedy Space Center in Cape Canaveral, Florida, where only light boxes can be used, and the light intensity was 1500 to 3000 lux.

I hired Karen T. Stewart, PhD, and trained her to help make the light/dark/sleep schedules. Figure 2 shows one of the schedules that we made. We had the astronauts go to bed 1 hour later each day and be exposed to bright light in the evening and before bed for 3–5 hours per day to help their circadian rhythms delay. The schedule also shows them to avoid bright light after waking, because light at that time would advance their circadian rhythms and inhibit the desired delay. We gave them a set of dark sunglasses, with different amounts of light transmission, to wear when it was absolutely necessary to go outside during daylight at a time when bright light was to be avoided. We were fortunate that the bright lights on the crew quarters ceiling and the light boxes at Cape Canaveral were already set up by Czeisler’s team.

Another one of our schedules can be seen in Stewart and Eastman [21]. This also had a gradual delay of sleep with bright light before bed to facilitate the delay. In this case, we also included bright morning light after waking on the 2 days before the launch. This team of astronauts was required to follow a gradually advancing sleep schedule after launch, while on orbit, so we didn’t want their circadian rhythms to delay too far. In our night shift protocols [22, 23], we call this advancing light the “light brake.”

In contrast to the slam dunk kind of schedule that Czeisler used, we employed a gentler, nudging approach.

After our first mission, Dr. Jennings sent me a letter, which said “Dear Dr. Eastman: The STS-39 flight has concluded successfully. I want to thank you for your part in making this challenging mission a success. The crew’s comments about the light therapy have been very positive during the preflight period, while on orbit, and since return.”

On the day of the launch of our first mission, I was working at home, as usual, and was about to leave to go to the lab, when the mission was scrubbed (postponed). They wanted us to immediately make a new schedule of light and sleep for the astronauts who had to wait a few more days for the next attempted launch. We were not prepared for this, and I stayed home to work on it. When we were done, I was about to leave the house when I saw something weird. The ceiling in our living room looked like it was pulsating, wobbling, moving! Was I having a hallucination? I remembered that workers had been putting a new roof on our row of townhouses, but they left when it started to rain. They were driving away, and I yelled out to them. The boss man came back, took one look at the ceiling, and said—Give me a screwdriver and get a bucket! He stood on a chair, made a hole in the ceiling with the screwdriver and water came gushing out. I won’t bore you with more details. The point is that if the mission had not been scrubbed, then the damage to our house would have been much worse.

Some of the workers in NASA’s mission controls also had to be night shift workers during the space shuttle missions because they needed 24 hours coverage. Karen and I made schedules for the night shift workers at the Marshall Space Flight Center in Huntsville, Alabama. One schedule can be seen in Stewart et al. [24]. Bright light before bed and a gradually delaying sleep schedule were used until sleep time occurred during the day allowing for good sleep after the night shifts which were from midnight until 8:00 or 9:00 am. There was a light brake for several days near the end of the mission, to maintain the current phase position. When the mission was over, we used a gradual delay of sleep with bright light before bed to shift them back to sleeping at night.

Karen and I made schedules for 10 space shuttle missions, and then she got a job at NASA where she continued to make the schedules.

## Space Wars

For my first studies, I didn’t have a lab or any research assistants. So, I had the subjects help me carry light boxes to their homes and help me put black plastic on their bedroom windows. I still lived in Hyde Park close to the University of Chicago campus. It was convenient to get U of C students as subjects, and they came to my apartment for brief visits, instead of Rush which was miles away. My perfect subjects were ABDs, or All But Dissertation, and U of C was famous for having lots of them. They were finished with classes and were free to sleep at any time, which was perfect for my studies.

At some point, I actually got some space at Rush. It was in an old building, Schweppe, now demolished. You took the elevator up to the 13th floor and then walked upstairs to the 14th floor. There were two rooms with pipes hanging in the ceiling. One room was my office, and the other was for my research assistants. I was thrilled. I named it the Biological Rhythms Research Lab. As my research grew and I needed more space, I decided to give up my office to have more space for the research. Cartwright managed to find a place for me in a shared office in a building across the street. I would walk across the street, or through an underground tunnel to go to the lab. It became more convenient to just call the lab or fax something over there than to walk there. I realized that I could be anywhere and have this kind of communication with the research assistants. This was the start of my working from home, which ended up being the way my life progressed, except now it’s even better because we have email and Zoom. When the pandemic came, I was already set up at home. I sit here now typing on a computer in a very large office, which would be the master bedroom in a normal house, surrounded by four big file cabinets, three printers, a scanner, and a copy/fax machine.

The “space wars” went on for a long time. Al Rechtschaffen suggested that I rent some office space and pay out of my direct costs. So, I rented several offices in the Hyde Park Bank Building and paid the rent and phones out of direct costs. We made one room into a place for giving subjects bright light while sitting around a big round table. Another room was made dark, with two Lazy Boy recliners for collecting saliva samples to be analyzed for melatonin. Starting with the SDN study [15], all studies were run in the Hyde Park lab until the year 2000. I didn’t rent an office for me there; I worked at home. The 14th floor Schwepe lab was gone. I was always asking Cartwright for more space.

After many years of having the lab at the Hyde Park Bank Building, someone figured out how unusual it was because Rush got the indirects from my grants. The indirects should have paid for my rent. Eventually, they (Rush) started paying the rent. In the year 2000, they built a big 3379 sq ft lab for me. I suppose Cartwright helped make that happen, or they just got tired of paying rent to the Hyde Park Bank Building. So, for many years, I did not feel supported by her enough to get space at Rush. My first NIH grant was in 1986, so it was 14 years before I got enough space at Rush. I learned that space is even more valuable than money.

One of the best times of my life was helping to design the new lab. It had two bedrooms (now three), a big “night shift” room for delivering bright light to subjects with three computers for performance testing, a big lazy boy room with three lazy boys (now six) for collecting saliva samples to be analyzed for melatonin, a kitchen with a big freezer, microwaves, dish washer, etc., for preparing food for subjects who lived in the lab, a bathroom with a shower (now two bathrooms), and eight offices with one for me! But you know what they say about teaching old dogs new tricks. I was so used to working at home that I still worked at home a lot of the time. When it came to reading papers and writing papers and grants, I had to be at home. I would say to the people in the lab, I’m not coming to work today. I have too much work to do. And they understood what that meant. I went to the lab for journal clubs, to work on budgets, to interview people to hire, etc.

The new big lab enabled us to do better studies. In several studies, supported for eight years by an R01 from the CDC/NIOSH, subjects came to the lab for many consecutive night shifts. These studies included NSW (Night Shift Work) [25, 26], ZAP [22, 27, 28], and more.

## Winter Depression, Seasonal Affective Disorder, and the Placebo Effect

In the 1980s, Al Lewy [29], Norman Rosenthal [30], and others were using light boxes to treat seasonal affective disorder (SAD). I had light boxes. I studied human rhythms. This was a seasonal rhythm. I wanted in! The problem was that there was no good placebo for bright light. Dim light was used as a placebo control in the first studies, but since then the media was full of stories about bright light, which means patients would expect the bright light to work better than the dim. In fact, even in the first studies, patients thought that the bright light would work better [30]. If patients think that one treatment will work better than another, if they have higher expectations for one, then the placebo effect, which is a big component of all antidepressant treatments, will be larger, and any difference in antidepressant response between bright and dim light could be entirely due to the difference in placebo effects. Some researchers used evening light as the placebo for morning light. Although evening light may be plausible to patients, many researchers found that evening light was as good as morning light and, therefore, not inert.

I was moping around the house complaining that I could never devise a good study, because there was no good placebo for bright light. My boyfriend, Larry Chait, PhD, a behavioral pharmacologist, tried to comfort me, or maybe just wanted to shut me up. He said—Let’s see—You need something similar to a light box, some weird new-age electrical device (light boxes were new at the time) that will emit a natural environmental factor which travels through the air to impinge upon the person and make them feel better. How about a negative ion generator? It turns out that there was a large scientific and popular literature about how negative ions can improve mood, energy, and affect serotonin transmission, and that our exposure to negative ions varies seasonally. I thought it was such a good idea that I married him!

**Figure 3.**
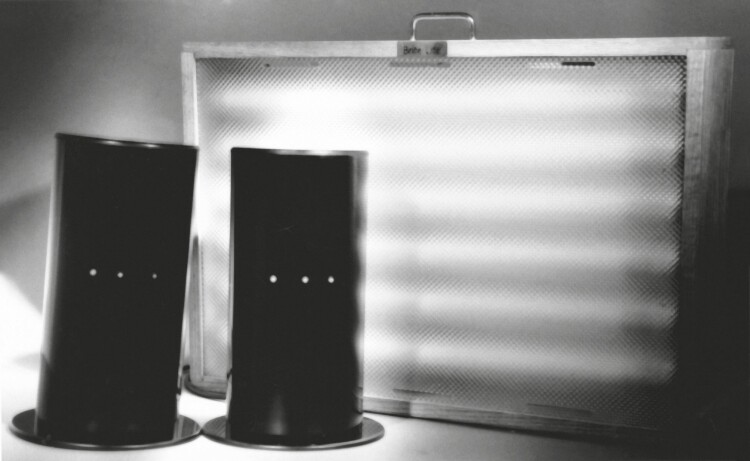
Photo of the light box and negative ion generators used in the winter depression study that we published in 1998 [31].

We used “deactivated” negative ion generators as our placebo control. I obtained an R01 from NIMH for 9 years of funding. Our final study [31] compared morning light, morning placebo, and evening light. Figure 3 shows the light box, which was 43.5 cm tall, and the negative ion generators which were 32 cm tall. We built these impressive looking ion generators, with white noise generators inside. The shiny black cylinders had three small lights on the front that flashed between green and red. I demonstrated their use to each patient assigned to them and gave them fussy detailed instructions for use. The patients had to set them up on a desk or table, one on each side of them. They used a ruler that we provided to place them 15 inches (38.1 cm) apart with 15 inches between the patient and each generator. I was equally fussy about the light box, which had to be set up exactly 15 inches in front of them. It produced about 6000 lux. Before the baseline week, patients were given information packets promoting bright light and negative ions. At the end of the baseline week, after patients had seen their equipment turned on, they completed an expectation questionnaire. We achieved our goal of having no difference in expectations between the treatments, and we had given them plenty of time to read and think about it.

Michael Young, PhD, was a terrific collaborator. He ran the diagnosis and depression ratings part of the studies and having offices in another building helped keep the double blind.

We only analyzed data from the “deactivated” negative ion generators. Actually, there was only one set of cylinders that actually had a negative ion generator inside and having that allowed me to say truthfully in the consent form that they might get active or deactivated generators.

**Figure 4.**
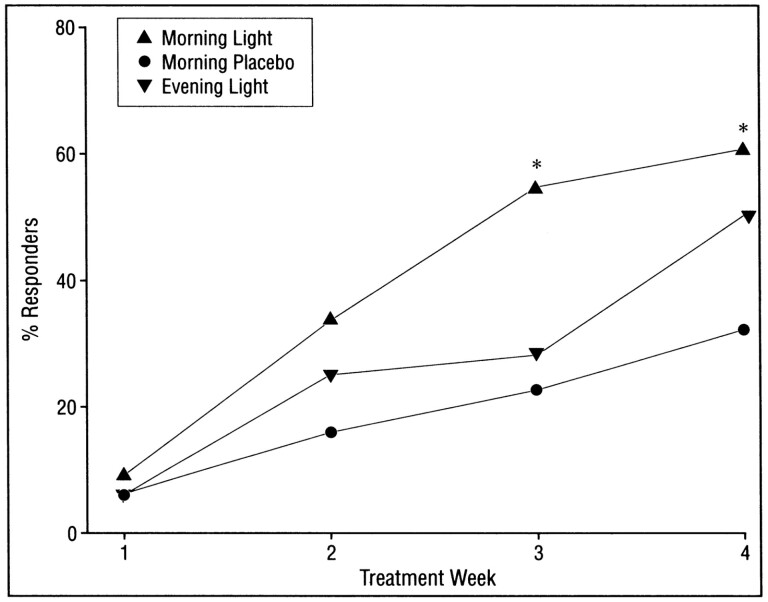
Percent of patients with nearly complete remissions, defined as achieving both a 50% decrease (from baseline) in the 24-item Structured Interview Guide for the Hamilton Depression Rating Scale, Seasonal Affective Disorder Version (SIGH-SAD) and a score of 8 or lower. The asterisk indicates *p* < .05 compared with placebo. From reference [31] with permission.

Figure 4 shows the difference in depression ratings among the three groups. There was a significant difference between morning light and morning placebo by week 3. A large component of the antidepressant effect was due to the placebo effect, just like in antidepressant drug studies [32, 33].

I wasn’t prepared for what happened when I had to debrief my first patient who had a good response to the negative ion generator placebo. She came in all happy and bubbly, quite a change from when I met her 4 weeks previously to give her the ion generators and tell her how to use them. When I told her they were placebos, she was devastated. Even though she had been feeling so much better, she was now very upset. She could hardly believe it. I tried to ease her embarrassment and concerns, but I wasn’t very good at it. After that I developed a good little speech for those who responded to the placebo. I explained that they might have been helped by the fixed early wake up time, that all people are helped by the placebo effect, whether they are smart or not, that it’s good to have a response to a placebo, that part of all treatments are placebo effects. I can’t remember exactly—it was over 30 years ago.

In a retrospective book chapter published in 2001 [34], I listed the six studies, so far, that achieved the goal and presented data to show that patients did not have higher expectations for light than placebo. Four used bright vs. dim head mounted light visors. One used a light box that was turned off but hummed, and patients were told that it was emitting light that could not be seen, infrared light. Our study was the only one that showed that light treatment was better than a placebo. The main point of the paper was that good placebos were still needed for light treatment studies of depression. It turns out that if you write too truthfully about placebo effects, you’re not very popular with clinicians or researchers who employ bright light treatment.

When I was starting the winter depression studies, Al Lewy asked me to write a paper on placebo effects for the Psychopharmacology Bulletin. I protested, but he insisted because I was using an unusual placebo for light treatment. I thought I knew what placebo effects were, but after doing a literature search, I was shocked at what I found. My work on that paper [35] greatly affected the design of our research. Looking back on my career, I think that it is one of the most important papers that I wrote, and it is still relevant today. In a meeting in 1995, Aschoff was holding a copy of that paper and looking for the author who was me! Afterwards, he sent me a letter that said, “Two days ago I could listen to a lecture on ‘Placebo effects’ by the Pharmacologist Habermann (GieBen); He was half as clear as you in your enlightening article – I gave him a copy.”

Recently (December 2, 2022), this paper was cited in a CNN Health article. One sentence was copied exactly. I had written about the bizarre treatments that had been used in the history of medicine such as bleeding, crocodile dung, ground Egyptian mummy, and more. I wrote “The enormous power of the placebo helps explain why physicians continued to be useful, respected and highly honored members of society despite the painful, abhorrent, unscientific and often dangerous treatments they prescribed” [35].

## Katie Sharkey and Melatonin

Katie was in the MD, PhD program at Rush University Medical Center. For the PhD part, she had the choice of being Cartwright’s last graduate student or my first. Luckily for me, she chose me! We wrote an NIH grant together, Melatonin Human Circadian Rhythms and Sleep, but it was not funded. They didn’t like, or maybe didn’t understand, our measure of circadian phase, which was rectal temperature with mathematical demasking, and they mentioned saliva sampling as a better measure. Katie borrowed a centrifuge, and other equipment and ran three subjects in her apartment to get saliva samples analyzed for melatonin. The subjects were sprawled all over on different pieces of furniture and cushions. She got nice melatonin profiles and with that we resubmitted and got the R01 for 4 years. She also got an NRSA F31 for a stipend. These grants supported the two studies in her dissertation, KAT [36] which was run in the Hyde Park lab and PRO [37] which included PSG and was run in the hospital. We made one of the rooms in the Hyde Park lab into a “lazy boy room” with two recliners to collect saliva samples in dim light. When subjects had to go to the bathroom, we wheeled them down the hall in a wheelchair while the subjects wore very dark goggles.

Once we started measuring circadian phase with melatonin profiles and the dim light melatonin onset (DLMO), we always ran all our studies with that measure. It helped us get more grants and changed the lab forever. I can’t think of a bigger gift a student could have given me.

Katie got her two degrees (MD and PhD) and then did her residency with two specialties (psychiatry and internal medicine) and had two children. I guess she liked to do everything in twos.

## Light History and Light Sensitivity

As a child, I was very sensitive to light. I remember being woken up in the morning and screaming when they turned on the light because it was so bright, practically blinding. In grade school we all had to read an eye chart. If it was very bright in the room, they would send me to the eye doctor (probably an optometrist), but if dim enough I could read it OK. He said that my lens would get darker with age, and that until then I could wear sunglasses in class. I didn’t do that—it would have made me a freak! Sunglasses were not “in” then. I am able to see better than other people in dim light. I realized how big the difference was when going to the movies with two friends. I was walking down the aisle in the theater, and they were lagging behind. I looked back and they were staring at the floor. I thought that they had lost a contact lens or something else small. I walked back to help them. It turns out that they just couldn’t see well enough to walk forward. Even now, I have to wear sunglasses outside if it’s sunny, I can’t drive without sunglasses if it’s sunny, and I hate very sunny weather. I don’t like bright light, which is pretty ironic given my research path.

Marc Hebert came to the lab in 1997 to do a postdoc with me. He was also interested in light sensitivity. We devised a study to see if people who were exposed to a lot of bright light became less sensitive to light, and if people who were in dim lighting a lot became more sensitive to light. This study was run in the Hyde Park lab. We used melatonin suppression from light in the middle of the night as our measure of light sensitivity. We used the lazy boy room and method for getting melatonin profiles that Katie had developed. Thank you, Katie! We collected saliva samples every 30 min and had them analyzed for melatonin. The subjects were in dim light (<15 lux) during saliva sampling except for 3 hours of bright light (500 lux) in the middle of the night. Each subject lived for a week getting as much bright light as possible. We brought light boxes to their homes or labs or work places and asked them to get at least 4 hours of bright light each day by going outside and/or using the light boxes. During the dim week, we asked them to stay indoors as much as possible and gave them very dark welders’ goggles to wear if they had to go outside. There was more suppression of melatonin after the dim light week than after the bright light week. Previous light history could affect one’s sensitivity to light. This study, CPG [38], is one of my most often cited publications.

This paper started a new line of research for several people [39–41]. The first one after ours was from Czeisler’s lab [42] and came out in 2004. They compared melatonin suppression to 6.5 hours of 200 lux at night after subjects spent 3 days in the lab in very dim light (0.5 lux) and after they spent 3 days in the lab in 200 lux. Just like in our study, there was more suppression after dim days. But they didn’t cite our 2002 paper. I sent a brief email to Czeisler asking why not. He called me and was very apologetic. He said that their paper had gone through many submissions and rejections and rewriting, and it was an accident that our paper didn’t get cited. The first author sent me a long email also apologizing.

## Ultradian LD Cycles, Phase Response Curves, and Circadian Period (tau)

I saw a poster at a Sleep Meeting that really impressed me. I think that it was by Shawn Youngstedt, PhD, at APSS 2002 [43]. I’m not sure that was the meeting, but I am sure that it gave me an idea for a new study. I remember drawing the protocol diagram on the plane on the way home. I was very excited! The poster showed a new way to make PRCs to bright light. Their subjects were put on a 90-min day with 30 min for sleep in the dark and 60 min awake in room light. Circadian rhythms free-run in this protocol, because they can’t entrain to such short LD cycles. Each subject was exposed to 3 hours of bright light (3000 lux) for 3 days at one time of day across the 24 hours. Different subjects were exposed to the bright light at different times of day. Circadian phase was determined from urinary melatonin before and after the three bright light days. The magnitude of the phase shifts vs. the time of day the bright light was applied produced the PRC. The protocol is best seen in figure 1 of their paper [44].

This was the start of the LAM studies (Light and Melatonin). I drew a protocol to make a melatonin PRC. I made it a 4-hour day, 1.5 hour for sleep in the dark and 2.5 hour awake in room light. There were three 24 hours days of this ultradian protocol. Each group of three subjects would get a pill at one of the six wake times across the 24 hours. Before and after the ultradian LD cycles, we would measure circadian phase with 30 min saliva samples for melatonin. This 5-day protocol was repeated, with 9 days in between. In one session, the pill would be 3 mg melatonin, and in the other, it would be a placebo pill. The placebo session would show how much the circadian rhythms delayed; it would show the free-running period. The other session would show how melatonin changed it, i.e. the phase shift due to melatonin plus the phase shift from the ultradian protocol. To get the phase shift just due to the melatonin, we would subtract the phase shift from the placebo session from the phase shift during the melatonin session. The protocol can best be seen in figure 1 of one of our papers [45].

We ended up using this protocol to make PRCs to 3 mg melatonin [46], 0.5 mg melatonin [45], bright light ~3500 lux [47], blue light from little GoLites [48], and a PRC to bright light in adolescents [49]. We used a slightly different ultradian LD cycles for the adolescents, 2 hours of dark/sleep and 2 hours awake in dim light, so they could get more sleep.

We also wrote about the free-running periods that were derived from the placebo or no bright light sessions [50, 51].

## Out of Africa—Ancestry

During a meeting of Mark Smith’s dissertation committee, one member was commenting on the individual differences in phase shifts due to light exposure from one of the studies. We were so used to seeing different amounts of phase shifts in different subjects put on the same protocol that we didn’t really think about it anymore. This committee member, Shunbin Xu, MD, PhD, asked if there were racial differences that might account for some of the variability. Mark went back to his data on phase shifting with gradually shifting sleep schedules and bright light [52, 53] and our data in which we calculated tau [50] and found differences between black and white participants. Black people had shorter free-running periods and phase advanced more in the phase advancing protocol and less in the phase delaying protocol. We followed up with a bigger sample size [54]. This little remark at Mark’s dissertation committee meeting led to a whole new area of research for us.

In addition to starting us on this new line of research, Mark was probably the most productive graduate student anybody could have. He worked on the JET and ZAP studies and was first author on 10 publications [22, 23, 27, 28, 52, 53, 55–58] and an author on several others.

We did two studies, the PAT studies (Phase And Tau), in which we calculated circadian period (tau) from an ultradian LD cycle and then measured the phase shift to a 9 hours advance of the sleep schedule, known as Chicago to Kenya [59] or a 9-hour delay of the sleep schedule, Chicago to Japan [60]. Each subject was put in the ultradian LD cycle (LD 2.5:1.5 or LD 3:2) and then the phase shifting protocol and lived in the lab for about 2 weeks. We obtained circadian phase and phase shifting data from the same subject, instead of having to compare groups of subjects in one study with groups from another study. We used buccal (cheek) swabs to collect DNA samples which gave us the percent European and the percent Sub-Saharan African for each subject to make sure we were getting people who were mostly one or the other.

The European-Americans had longer free-running periods and delayed more when the sleep schedule was delayed. When the sleep schedule was advanced, some of the European-Americans phase delayed instead of advancing; their circadian rhythms shifted the wrong way. There were correlations between the free-running period and the magnitude of the phase shifts; those with longer free-running periods phase delayed more and those with shorter free-running periods phase advanced more. I speculated that when humans lived in Africa around the equator, they had periods closer to 24 hours, and when some humans migrated out of Africa to more northern latitudes, longer periods evolved. There are complicated theories about why a longer period helps with the changes in photoperiod with seasons. But in our early-bird dominated society, it’s better to have a shorter free-running period.

**Figure 5.**
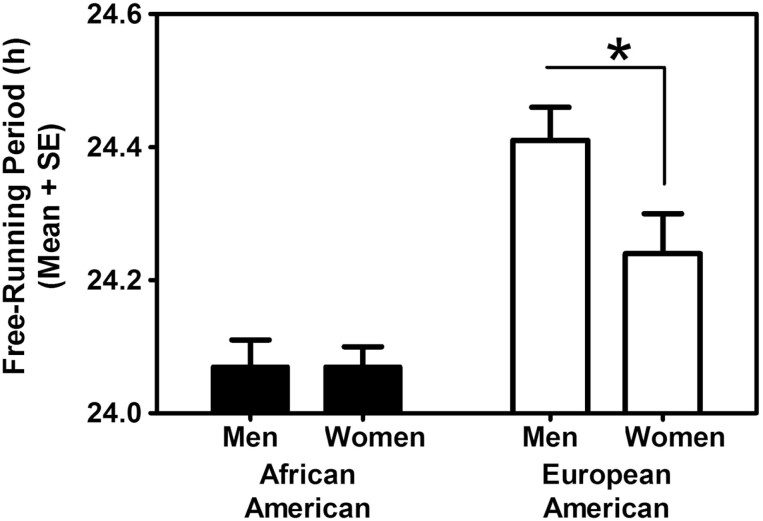
The free-running periods (tau) derived from an ultradian light/dark and wake/sleep cycle. The asterisk shows one of the significant differences (*p* < .05). From reference [61] with permission.

We combined the data from the two PAT studies, and a figure from that analysis [61] is shown here. Figure 5 shows that the free-running periods are shorter in African-Americans than in European-Americans. It also shows that there are sex differences, with men having longer periods than women, but only for the European-Americans.

## Stephanie Crowley—Teenagers in the Lab

Stephanie came to work for me as a research assistant just when we were moving from the Hyde Park lab to the new big lab. She helped us with the move. She was the 34th research assistant that I had hired, and she ended up being more like a graduate student or postdoc than a research assistant. She helped run the studies from the NSW grant funded by the CDC/NIOSH which started in the new lab, and she ended up being first author on two papers from that grant [25, 26]. She helped me write the renewal of that grant which we called ZAP, and we got it for five more years. I remember her practically sleeping in one of the bedrooms which was not in use while helping me write that grant. I don’t remember if she actually slept there—you’ll have to ask her, but the point is that she was a hard worker. She earned authorship on four other publications from our lab [62–64, 55].

After 3 years as a research assistant, she went to Mary Carskadon’s lab to get a PhD. Six years later, I was able to coax her back as an assistant professor. She worked on studies from the JET grant [65, 66] and the PAT grant [59–61] while starting her own research program on adolescents. She had to add many new procedures to the lab to be able to run these teenagers, and when the pandemic came there were even more adjustments to be made to be able to continue to run these studies. She has secured 4 R01s as PI to date and is running the lab. All I have to do is comment on any papers she writes from these grants, which I helped write. My life is so much easier. No more administrative, bureaucratic stuff, just science. I am semi-retired, and Larry and I bought a casa in Mexico.

## Who Did I Leave Out?

This has not been a review paper, but rather a bunch of stories. There are postdocs and research assistants and others who made important contributions to our research but were not mentioned. Helen Burgess started as my postdoc and worked her way up to Full Professor before leaving Rush to hopefully have a better life. We wrote some grants together such as SAL (Short And Long) and DAH (Dlmos At Home), and we worked well together for about 10 years. Vikki Revell was a postdoc who is probably the smartest person to work in our lab, and my favorite person to write with [47, 48, 64, 67–69]. There were some research assistants who were more like graduate students and earned first authorship on papers. Erin Baehr was first author on my most cited paper [70]. Stacia Martin also earned first authorships [18, 71]. Christina Suh [59], Vicky Tomaka [59–61], Tom Molina [45, 48, 54] all made important contributions. Ieva Misiunaite is still there, is first author on a recent paper [72], and helps Stephanie run the lab.

## Notes

This paper is part of the Living Legends in Sleep Research series, which is sponsored by Idorsia Pharmaceuticals and Jazz Pharmaceuticals.
